# Schizophrenia trials conducted in African countries: a drop of evidence in the ocean of morbidity?

**DOI:** 10.1186/1752-4458-6-9

**Published:** 2012-07-06

**Authors:** Marianna Purgato, Clive Adams, Corrado Barbui

**Affiliations:** 1 Division of Psychiatry, University of Nottingham, Nottingham, UK; 2 Department of Public Health and Community Medicine, Section of Psychiatry, University of Verona, Verona, Italy

**Keywords:** Clinical trials, Africa, Schizophrenia

## Abstract

**Objective:**

To quantify schizophrenia trialling activity in African countries and to describe the main features of these trials.

**Methods:**

We searched the Cochrane Schizophrenia Group Register, which contains 16,000 citations to 13,000 studies relating only to people with schizophrenia or schizophrenia-like illness, to identify schizophrenia trials conducted in Africa without time limitation.

**Results:**

A total of 38 trials met the inclusion criteria and were included in our analysis. Of the 54 countries of Africa, only 8 produced at least one trial: South Africa produced the majority of trials (20 out of 38 trials, 53%), followed by Nigeria (7 out of 38 trials, 18%) and Egypt (4 out of 38 trials, 11%). The majority of studies investigated the efficacy of pharmacological interventions, were short in duration, and employed a double-blind design. The quality of reporting was generally poor. We found six trials comparing antipsychotics from the WHO Essential List of Medicine versus new generation antipsychotics. In terms of efficacy and acceptability, these studies failed to show any advantage of newer antipsychotics over first-generation agents.

**Conclusions:**

We observed an impressive mismatch between the number of individuals with schizophrenia living in African countries, estimated to be around 10 million, and the overall number of patients included in African trials, which is less than 2,000. These few trials were of low quality and appeared not to reflect the real needs of the population. We argue that the concept of pragmatism should be introduced into the design of randomized trials in African countries. Pragmatic trials should investigate whether treatments, given in real-world circumstances, really have clinically meaningful effects.

## Introduction

Most of the global burden of mental illness falls to the poorest nations, where 80% of world’s population live [1]. Africa is the second-most-populated continent, with around 1 billion people, or 15% of the world's population. For schizophrenia, a major psychiatric disorder that affects about 0.5% of world population [2], it has been estimated that between 4 and 5 million individuals afflicted by these severe psychiatric disorders live in African countries [2]. Despite these impressive numbers, the worldwide deficit of research about psychiatric disorders is particularly acute in low- and middle-income countries (LAMICs), including several African countries [3]. LAMICs devote less than 1% of their health expenditure to mental health and have poorly developed mental health policies and research infrastructures [4].

For patients, carers and policymakers local data are important. Even well-conducted trials, if undertaken in a very dissimilar care-culture may be difficult to apply. Local trials are important and informative whether they agree or disagree other similar studies from afar. All evidence must be considered but the local perspective not ignored. The quality of any trialling activity is another crucial issue, as it may vary across nations [5,6]. For example, the volume and quality of trial research from China has been considered in many surveys and quality remains a major concern [7-11]. Elsewhere it has been shown that pioneering mental health trials from LAMICs are of as mixed quality as their more accessible counterparts from richer nations, but cannot be identified in commonly used bibliographic databases [12].

The Cochrane Schizophrenia Group produces and maintains a register of all studies [13]. This involves regular and systematic searching of 71 databases worldwide. The studies identified in this way are reliably indexed by country.

The aim of this work is to quantify schizophrenia trialling activity in African countries and to provide detailed information on the main clinical and methodological features of these trials. Our analysis could be used by researchers and policy makers to help plan future research to cover the mental health needs of African population.

## Methods

### Search methods for identification of studies

We searched the Cochrane Schizophrenia Group Register to identify any trial conducted in Africa without time limitation. The Cochrane Schizophrenia Group Register contains 16,000 citations to 13,000 studies relating only to people with schizophrenia or schizophrenia-like illness [13]. The register includes all published and unpublished references to randomized, quasi randomized and controlled clinical trials without language restrictions. These studies are indexed regarding the country of origin, the interventions under study and the number of participants. The last version of register was February 2012.

### Types of studies

We included all randomised controlled trials and controlled clinical trials undertaken in any African country. Included studies compared any pharmacological and non-pharmacological treatments with other active treatments or placebo. Only studies that enrolled patients in Africa were considered. Multicentre studies were included only if all centres enrolling patients were located in Africa.

### Types of participants

Participants were in- and out-patients of both sexes, with a primary diagnosis of schizophrenia and related psychotic disorders, according to the criteria described in the DSM, ICD, or according to any other clinical or standardized criteria.

### Selection of trials

Included and excluded studies were collected following the Preferred Reporting Items for Systematic reviews and Meta-Analyses – PRISMA flow diagram [14]. From the Cochrane Schizophrenia Group Register we extracted all records corresponding to studies carried out in Africa. We examined all titles and abstracts, and obtained full texts if the word “random” or “randomised” or “control” or “controlled” was present in the title and/or abstract. MP and CB read the full texts, determined whether they met inclusion criteria and extracted the data. Considerable care was taken to exclude duplicate publications.

### Assessment of risk of bias in included studies

For quality assessment we used the Cochrane risk-of-bias tool (Oxford, England, Cochrane Collaboration). This instrument consists of seven items [15]. Two of the items assess the strength of the randomization process in preventing selection bias in the assignment of participants to interventions: adequacy of sequence generation and allocation concealment. The third and fourth items assess the influence of performance bias and detection bias on the study results. The fifth item assesses the likelihood of incomplete outcome data, which raise the possibility of bias in effect estimates. The sixth item assesses selective reporting, the tendency to preferentially report statistically significant outcomes. This item requires a comparison of published data with trial protocols, when such are available. The final item refers to other sources of bias that are relevant in certain circumstances, such as, for example, sponsorship bias.

### Data extraction

The following information was extracted using an electronic spreadsheet: year of publication, geographic area (country of Africa), type of experimental and control intervention, sample size, weeks of follow-up, diagnostic criteria, number of outcome measures, Gross Domestic Product (GDP) at the time when each study was carried out. For studies that compared antipsychotics included in the WHO Essential List of Medicines [16] with new generation antipsychotics we additionally extracted efficacy and acceptability data. For efficacy data, the mean change from baseline to endpoint, or the mean scores at endpoint, at the Brief Psychiatric Rating Scale (BPRS) or Positive and Negative Syndrome Scale (PANSS) were extracted, together with the standard deviation (SD) or standard error (SE) of these values, and the number of patients included in these analyses. For acceptability data, the number of patients leaving the study early for any reason was extracted.

### Data presentation

We calculated simple percentages (%). To ascertain whether sample size and number of efficacy measures have increased in the last 40 years, we used a box plot diagram and a nonparametric test for trend (extension of the Wilcoxon rank-sum test). For each study that compared antipsychotics included in the WHO Essential List of Medicines with new generation antipsychotics, efficacy and acceptability data were entered and analyzed using the Cochrane Collaboration’s Review Manager software version 5.1 (Oxford, England, Cochrane Collaboration). Continuous data were analyzed using standardized mean differences (SMDs) with the random-effects-model (with 95% confidence intervals [CI]); for dichotomous outcomes, the odds ratio (OR) was calculated based on the random effects model (with 95% CI). This approach was used to present the results of individual studies, but no overall treatment estimates were calculated.

## Results

### Characteristics of included studies

**Figure 1:**
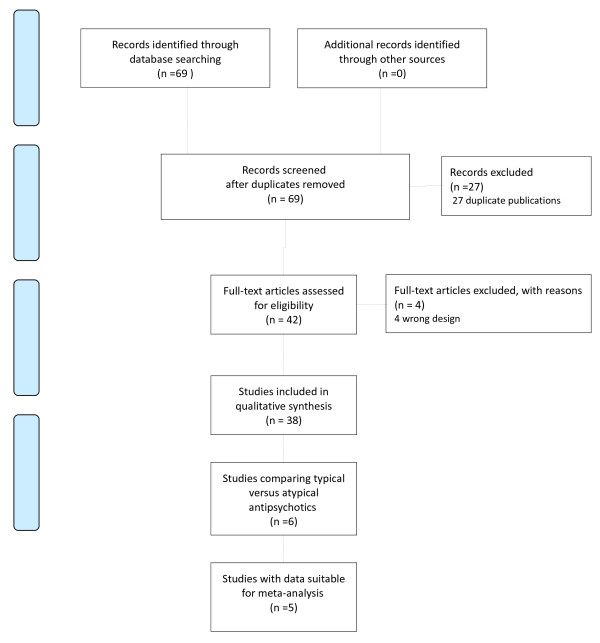
PRISMA Flow diagram.

The original search yielded 69 records, of which 27 were excluded because ineligible or not relevant. The remaining 42 records were assessed for eligibility. Thirty-eight trials met the inclusion criteria and were included in our analysis (Figure1) (for references of included studies, see reference list from [17-54]).

**Figure 2:**
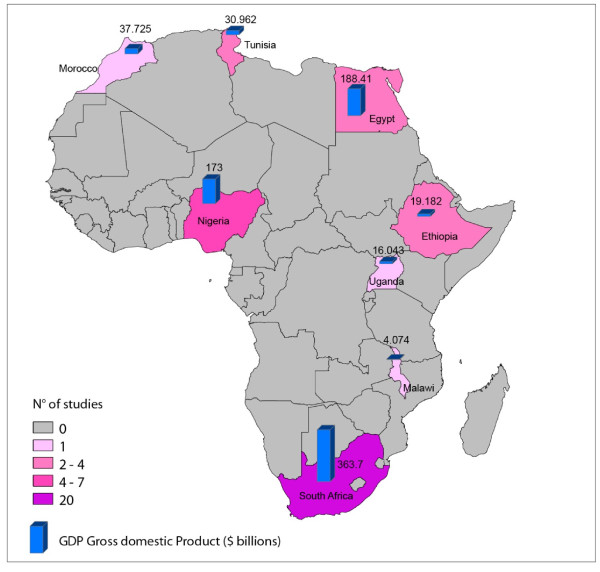
Distribution of schizophrenia trials by African country and Gross Domestic Product.

Eight out of the 54 countries of Africa produced at least one trial (Figure2). South Africa produced the majority of trials (20 out of 38 trials, 53%), followed by Nigeria (7 out of 38 trials, 18%) and Egypt (4 out of 38 trials, 11%). These countries had the highest Gross Domestic Product compared with the other countries producing trials in Africa (Figure2). The characteristics of all included studies are presented in Table1. The majority of studies investigated pharmacological interventions, with a drug *versus* drug design in most cases (33 out of 38 trials, 87%). Most studies were short in duration (22 out of 38 had a follow-up ≤ 12 weeks, 58%), had a double-blind design and used standardized diagnostic criteria (DSM or ICD). Local ethics committee approval has been mentioned in 22 out of 38 studies. Fifteen studies were funded by pharmaceutical industries, three studies were independent from drug industry and in remaining 20 studies the role of sponsorship was unclear (Table1).

**Table 1:** Characteristics of schizophrenia trials conducted in Africa

	**STUDIES (*n*** **= 38) No %**
**Type of comparison**	
Typical AP *versus* typical AP	12 (31.58)
Typical AP *versus* placebo	3 (7.89)
Typical AP *versus* no treatment	1 (2.63)
Typical AP *versus* atypical AP	6 (15.79)
Atypical AP *versus* atypical AP	4 (10.53)
Other pharmacological interventions	7 (18.42)
Non pharmacological interventions	5 (13.16)
**Year of publication**	
1970–1985	9 (23.68)
1986–2000	9 (23.68)
2001–2010	20 (52.64)
**Sample size**	
Min-20	4 (10.81)
21–40	12 (32.43)
41–60	10 (27.03)
61-Max	11 (29.73)
**Arms**	
Two arms	34 (89.47)
Three arms or more	4 (10.53)
**Weeks of follow-up**	
< 6 weeks	16 (42.43)
6–12 weeks	9 (24.24)
13–24 weeks	7 (18.18)
> 24 weeks	6 (15.15)
**Blinding**	
Open label	6 (15.79)
Single blind	7 (18.42)
Double blind	18 (47.37)
Unclear	7 (18.42)
**Diagnostic criteria**	
Unclear	9 (23.68)
DSM† − ICD††	25 (65.79)
Clinical diagnosis	4 (10.53)
**Number of outcomes**	
Min-5	13 (34.21)
6–10	14 (36.84)
11-Max	11 (28.95)
**Ethics committee approval**	
Unclear	16 (42.11)
Yes	22 (57.89)
**Sponsor (drug company)**	
Unclear	20 (52.63)
Yes	15 (39.48)
Independent study	3 (7.89)

**Figure 3:**
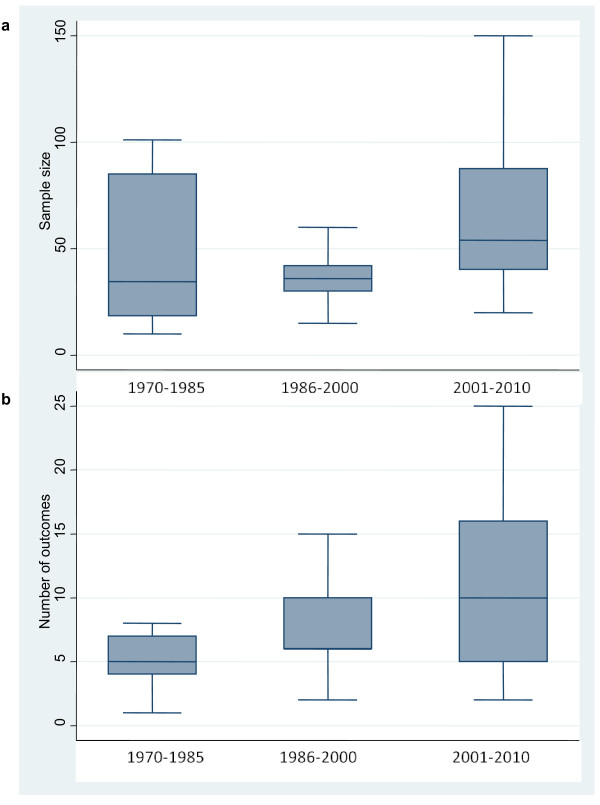
**Distribution of schizophrenia trials carried out in Africa by sample size (a) and number of outcome measures (b) from 1970 to 2010.** The horizontal line represents the median, the box extends to cover the interquartile range and the vertical line extends to the extremes.

While sample size only minimally increased over time (Figure3), as shown by Spearman's rank correlation coefficient (rho = 0.333; *p* = 0.043), the number of outcomes measures significantly increased over time (rho = 0.357; *p* < 0.027).

### Risk of bias

**Figure 4:**
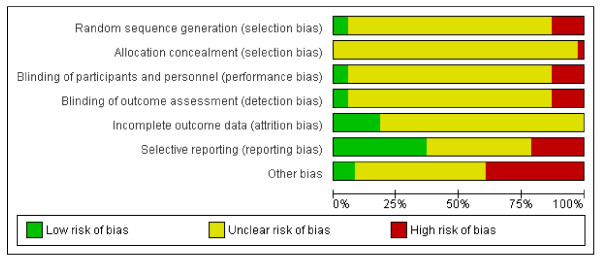
Methodological quality graph: review authors' judgements about each methodological quality item presented as percentages across all included studies.

The standard of reporting was generally poor (Figure4) (For study by study risk of bias, see Additional file 1). The generation of the randomization sequence and the concealment of allocation were not properly described in the majority of studies. Blinding was adopted in some studies, but not accurately described. The risk of other bias, such as sponsorship bias, could not be excluded, as several studies received financial support from pharmaceutical industries.

### Essential antipsychotics versus new generation antipsychotics

**Figure 5:**
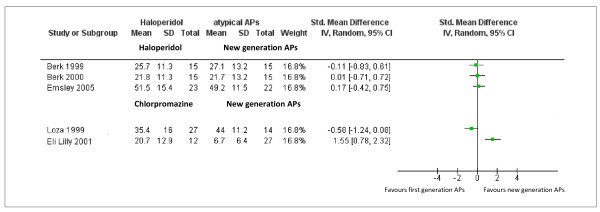
Efficacy data of schizophrenia trials conducted in Africa comparing antipsychotics included in the WHO List of Essential Medicines (haloperidol and chlorpromazine) with new generation antipsychotics.

**Figure 6:**
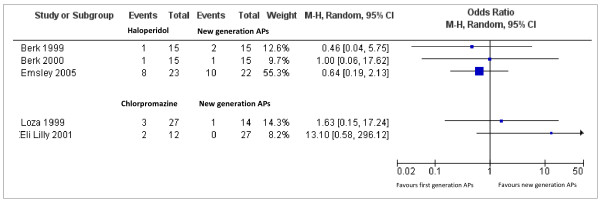
Acceptability data (total dropout rates) of schizophrenia trials conducted in Africa comparing antipsychotics included in the WHO List of Essential Medicines (haloperidol and chlorpromazine) with new generation antipsychotics.

Six trials compared antipsychotics from the WHO essential list of medicine versus new generation antipsychotics. Essential antipsychotics were haloperidol and chlorpromazine; new generation antipsychotics were quetiapine, olanzapine and risperidone. Efficacy and acceptability data, available for 5 studies only, are presented in Figures 5 and 6, respectively. In terms of efficacy, three studies comparing haloperidol versus new generation antipsychotics failed to show any difference. Studies comparing chlorpromazine versus olanzapine provided contrasting evidence. In one study chlorpromazine was less effective than olanzapine (SMD = 1.55, 95% CI 0.78 to 2.32 n = 39), in the other one chlorpromazine was slightly more effective than olanzapine (SMD −0.58, 95% CI −1.24 to 0.08 n = 41) (Figure5). In terms of acceptability, studies failed to show any difference (Figure5).

## Discussion

**Figure 7:**
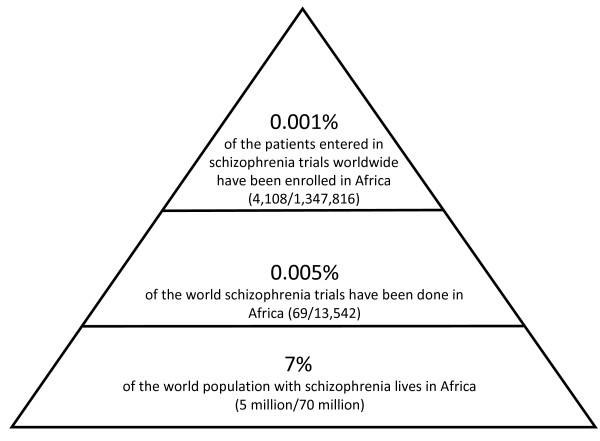
Pyramid describing the mismatch between the number of individuals with schizophrenia living in African countries and the number of trials conducted, and patients involved, in African countries.

To our knowledge, this is the first survey investigating content and quality of schizophrenia trials carried out in African countries. A first impressive result is the mismatch between the number of individuals with schizophrenia living in African countries, estimated to be around 5 million [2], and the overall number of patients included in African trials, which is less than 2,000 (accounting for 0.001% of patients included in the totality of trials conducted in schizophrenia at a global level according to the Cochrane Schizophrenia Register) (http://szg.cochrane.org/cochrane-schizophrenia-group-specialised-register). The mismatch is even more striking if one considers that 15% of the world population lives in Africa, but only 0.005% of the world schizophrenia trials have been conducted in the continent (Figure7). As one might expect, the great majority of trials on schizophrenia are conducted in high-income countries (32% in North America and 26% in Europe), but emergent countries like China, Brazil or India –with a middle-low or low Gross Domestic Product- are having a recent burgeoning of work [6]. For example, even if Chinese trials are often described as poor in methodological quality [9,55], China produced in the last decade a great amount of randomized trials (22% of all schizophrenia trials coded in the Cochrane Schizophrenia Group Register come from China) [6]. Also from Brazil and India there are examples of emergent high quality research activity [56].

A second issue is that these few trials appear not to reflect the real needs of the population. The World Mental Health surveys showed that more than 75% of those identified with serious mental disorders in LAMICs received no care at all, despite substantial role disability [57]. In sub-Saharan Africa, the treatment gap for schizophrenia and other psychoses has been shown to exceed 90%. Additionally, even where treatment is provided, it often is far below minimum acceptable standards. In such a situation it seems difficult to believe that trials addressing the efficacy of antipsychotic drugs versus placebo, or trials aiming to ascertain the added value of new generation antipsychotics over inexpensive “essential” antipsychotics can be seen as a priority. If one additionally considers that most trials were underpowered, failed to report basic methodological details such as, for example, information about the methods of random allocation, and its concealment from the study investigators, or how blinding was preserved, than the overall situation seems even more desolating, as the practical contribution of these studies to African patients, carers and policymakers is at least questionable. Unfortunately, trial quality has not substantially increased over time: while sample size increased only minimally over time, patient selection criteria and outcome assessments have become much more sophisticated, as suggested by the increase in use of standardized diagnostic criteria and by the steadily increase in the number of outcome measures. Although this trend may have increased the internal validity of findings, it has nevertheless allowed study of only highly refined groups of people with schizophrenia. This increasing drift from real world practice makes it difficult to apply trial results to typical patients [58]. Similarly, we observed that the number of outcome measures has increased during the last 40 years. This confusion of measuring suggests, at the very least, a lack of consensus on what is important.

Instead of increasing the drift from real world practice, randomized trials should be able to enroll real-world patients populations to be followed under real-world circumstances and assessed with outcome measures that are used in practice. As has already been happening in many other countries of the world, the concept of pragmatism should be introduced into the design of randomized trials in African countries. Pragmatic trials should investigate whether treatments, given in real-world circumstances, really have clinically meaningful effects. An example of this attitude is the series of TREC studies carried out in Brazil and India which randomized treatments given in day to day practice, left all subsequent decisions to clinicians, and used routine data as primary outcomes [59-62]. The concept of pragmatism could be similarly applied to the purpose of clinical trials, with a strong focus and commitment to answering questions of high public health relevance. Examples of trials undertaken with this logic are available, for example in the area of interventions for psychosocial wellbeing in humanitarian settings [63]. Another pragmatic area of interest is implementation science [64], with a focus on investigating the most effective interaction between specialist and non-specialist care providers, such as the extent to which tasks can be shifted and the duration, type, and frequency of training and supervision that are required. WHO has recently produced an evidence-based intervention package with recommendations to facilitate care at first and second level facilities by the non-specialist health care providers in low and middle income countries [65,66], but no randomized evidence exists on how this package should be implemented to maximize benefit at sustainable costs. Researchers and funders have tremendous responsibility in this context. Consortia and networks, advocacy organizations, universities and their partners should organize their activities considering that the implementation of a new generation of randomized trials in African countries is a pressing priority [64].

The present analysis has limitations. First, we did not include international multicentre studies where African countries were single sites, possibly loosing studies with good sample sizes and potentially better reporting. Second, despite the extensive searches, the possibility that some relevant studies have not been identified cannot be ruled out. The Cochrane Schizophrenia Group maintains a trial register by regularly and systematically searching 71 databases worldwide. However, it is possible that some journals are not indexed in these databases, and there might be some other local databases that are not included in our searches. Other factors are that some databases have become available only recently, and poor indexing within some databases may have impaired retrieval. We argue that accessibility to trials conducted in African countries should be improved by prospective registration of all future trials, as endorsed by the International Committee of Medical Journal Editors (ICMJE) [67] and the World Health Organization's International Clinical Trials Registry Platform [68].

In conclusion, this study generates information on the clinical and methodological characteristics of trials conducted in Africa on schizophrenia interventions. This information is needed to plan and implement future schizophrenia clinical trials, to suggest research areas that may have not received adequate attention, and to point to methodological aspects that need to be addressed when planning future trialling activities.

## Competing interests

The authors declare that they have no competing interest.

## Authors’ contributions

MP, CEA and CB designed the study. MP extracted data, MP and CB analyzed and interpreted data. MP and CB drafted the first manuscript. CEA commented and refined the manuscript in preparation for submission. All authors approved the final version to be published.

## Supplementary Material

Additional file 1 Risk of bias of included studies. Click here for file
