# Supplementary material for: Schizophrenia trials conducted in African countries: a drop of evidence in the ocean of morbidity?
Source: Int J Ment Health Syst. 2012 Jul 6;6:9. doi: 10.1186/1752-4458-6-9 (PMC3447718; doi:10.1186/1752-4458-6-9)
Supplement: Additional file 1 — Risk of bias of included studies. [file 1752-4458-6-9-S1.doc]

**Schizophrenia trials conducted in African countries:**

**a drop of evidence in the ocean of morbidity?**

Marianna Purgato^1,2^, Clive Adams^1^, Corrado Barbui^2^

*1. Division of Psychiatry, University of Nottingham, Nottingham (UK)*

*2. Department of Public Health and Community Medicine, Section of Psychiatry, University of Verona, Verona (Italy)*

**appendix 1**

**RISK OF BIAS OF INCLUDED STUDIES**

***Agara 2007***

| **Bias** | **Authors' judgement** | **Support for judgement** |
| --- | --- | --- |
| Random sequence generation (selection bias) | Unclear risk | Quote: "recruitment of patients into the study was done by random selection (...) the treatment and control group were allotted alternately". |
| Allocation concealment (selection bias) | Unclear risk | No information provided. |
| Blinding of participants and personnel (performance bias) | Unclear risk | No information provided. |
| Blinding of outcome assessment (detection bias) | Unclear risk | No information provided. |
| Incomplete outcome data (attrition bias) | Unclear risk | No clear information about data analysis. |
| Selective reporting (reporting bias) | Low risk | Outcomes data were reported. |
| Other bias | Low risk | Study was independent from pharmaceutical industry. |

***Anumonye 1976***

| **Bias** | **Authors' judgement** | **Support for judgement** |
| --- | --- | --- |
| Random sequence generation (selection bias) | Unclear risk | Quote: "A total of twenty-four adult chronic schizophrenics (...) were randomly assigned to one of two regimens". |
| Allocation concealment (selection bias) | Unclear risk | No information provided. |
| Blinding of participants and personnel (performance bias) | Unclear risk | Single blind. Quote: "The drugs were administered separately by a single-blind procedure (...) The size and shape of the tablets were not viewed as particularly important in very disturbed patients especially as there was no cross-over procedure involved and no patient knew about the test on the drug used by the members of the other group". |
| Blinding of outcome assessment (detection bias) | Unclear risk | No information provided. |
| Incomplete outcome data (attrition bias) | Unclear risk | No clear information about data analysis. |
| Selective reporting (reporting bias) | Unclear risk | Primary outcomes were reported. . |
| Other bias | Unclear risk | Drug companies were not mentioned (authors do not clearly declared that the study was independent). |

***Askar 1970***

| **Bias** | **Authors' judgement** | **Support for judgement** |
| --- | --- | --- |
| Random sequence generation (selection bias) | High risk | No randomized design. Quote: "Patients (...) were divided into a control, or placebo group, and a study, or drug group". (See the Cochrane review -Thioridazine for schizophrenia-, where this study was excluded because of non-randomized design). |
| Allocation concealment (selection bias) | Unclear risk | No information provided. |
| Blinding of participants and personnel (performance bias) | Unclear risk | No information provided. |
| Blinding of outcome assessment (detection bias) | Unclear risk | No information provided. |
| Incomplete outcome data (attrition bias) | Unclear risk | No information provided. |
| Selective reporting (reporting bias) | Unclear risk | No information provided. |
| Other bias | Unclear risk | No information provided. |

***Berk 1999***

| **Bias** | **Authors' judgement** | **Support for judgement** |
| --- | --- | --- |
| Random sequence generation (selection bias) | Unclear risk | Quote: "The patients were assigned randomly and consecutively to treatment". |
| Allocation concealment (selection bias) | Unclear risk | No information provided. |
| Blinding of participants and personnel (performance bias) | Unclear risk | Quote: "In a double-blind fashion". |
| Blinding of outcome assessment (detection bias) | Unclear risk | Quote: "The assessor was blind to randomized category". |
| Incomplete outcome data (attrition bias) | Unclear risk | Quote: "Data were analysed on a last observation carried forward basis". (In the results section the denominator was unclear). |
| Selective reporting (reporting bias) | Unclear risk | Scores and SD on rating scales were not reported - Brief Psychiatric Rating Scale (BPRS), Clinical Global Impression (CGI), Simpson Angus Scale (SAS), Barnes Akathisia Scale (BAS) - . Baseline investigations to assess urea and electrolytes, creatinine, thyroid function, full blood count, platelet count, liver function were not reported. |
| Other bias | High risk | Study was sponsored by Eli Lilly. |

***Berk 2000***

| **Bias** | **Authors' judgement** | **Support for judgement** |
| --- | --- | --- |
| Random sequence generation (selection bias) | Low risk | Quote: "The patients were assigned randomly and consecutively to treatment (...). Randomization was done by sequentially allocating patients to previously prepared medication kits according to allocated study numbers". |
| Allocation concealment (selection bias) | Unclear risk | No information provided. |
| Blinding of participants and personnel (performance bias) | Unclear risk | Quote: "double-blind". |
| Blinding of outcome assessment (detection bias) | Unclear risk | No information provided. |
| Incomplete outcome data (attrition bias) | Unclear risk | Quote: "Data were analysed on an intent-to-treat or last observation carried forward basis". (In the results section the denominator was unclear). |
| Selective reporting (reporting bias) | Unclear risk | Outcomes data were not clearly reported (no SD). |
| Other bias | High risk | Study was sponsored by Janssen Cilag. |

***Berk 2001***

| **Bias** | **Authors' judgement** | **Support for judgement** |
| --- | --- | --- |
| Random sequence generation (selection bias) | Unclear risk | Quote: "patients were randomized". |
| Allocation concealment (selection bias) | Unclear risk | No information provided. |
| Blinding of participants and personnel (performance bias) | Unclear risk | Quote: "double-blind". |
| Blinding of outcome assessment (detection bias) | Unclear risk | Quote: "double-blind". |
| Incomplete outcome data (attrition bias) | Unclear risk | Quote: "all data were analysed on a intent-to-treat or last observation carried forward basis". The denominator was unclear. |
| Selective reporting (reporting bias) | Low risk | Outcomes data were reported. |
| Other bias | Unclear risk | Sponsorship bias can not ruled out. |

***Botha 2010***

| **Bias** | **Authors' judgement** | **Support for judgement** |
| --- | --- | --- |
| Random sequence generation (selection bias) | Low risk | Quote: "Participants (...) were randomized using standardized tables". |
| Allocation concealment (selection bias) | Unclear risk | No information provided. |
| Blinding of participants and personnel (performance bias) | Unclear risk | No information provided. |
| Blinding of outcome assessment (detection bias) | Unclear risk | No information provided. |
| Incomplete outcome data (attrition bias) | Unclear risk | No clear information about data analysis. |
| Selective reporting (reporting bias) | Low risk | Outcomes data were reported. |
| Other bias | Low risk | This study seems to be independent from pharmaceutical industry. Authors declared no conflict of interest. |

***Brook 1998a***

| **Bias** | **Authors' judgement** | **Support for judgement** |
| --- | --- | --- |
| Random sequence generation (selection bias) | Unclear risk | Quote: "Random allocation to treatment". |
| Allocation concealment (selection bias) | Unclear risk | No information provided. |
| Blinding of participants and personnel (performance bias) | Low risk | Quote: "The study was a double blind controlled study (...) in order to blind the trial, independent clinicians were involved in the rating process and medication administration". |
| Blinding of outcome assessment (detection bias) | Low risk | Quote: "In order to blind the trial, independent clinicians were involved in the rating process and medication administration". |
| Incomplete outcome data (attrition bias) | Unclear risk | No information about data analysis. |
| Selective reporting (reporting bias) | High risk | Rating scale scores were not fully reported. SD were missing. |
| Other bias | High risk | Study was sponsored by Lundbeck. |

***Desta 2002***

| **Bias** | **Authors' judgement** | **Support for judgement** |
| --- | --- | --- |
| Random sequence generation (selection bias) | Unclear risk | Quote: "Subjects were randomized". |
| Allocation concealment (selection bias) | Unclear risk | No information provided. |
| Blinding of participants and personnel (performance bias) | Unclear risk | Quote: "double-blind (...) subjects were randomized to receive HCG 200 mg/day or an identically packaged inert placebo". |
| Blinding of outcome assessment (detection bias) | Unclear risk | No information provided. |
| Incomplete outcome data (attrition bias) | Unclear risk | No clear information about data analysis. |
| Selective reporting (reporting bias) | Low risk | Outcomes data were reported. |
| Other bias | High risk | Study was funded by Sanofi-Winthrop. |

***El Islam 1970***

| **Bias** | **Authors' judgement** | **Support for judgement** |
| --- | --- | --- |
| Random sequence generation (selection bias) | High risk | The term "randomization" is not used. |
| Allocation concealment (selection bias) | Unclear risk | No information provided. |
| Blinding of participants and personnel (performance bias) | Low risk | Quote: "Patients did not know that they were receiving different methods of ECT (...) Cases were alternately allocated to the unilateral and bilateral ECT groups by one of us, who was the only one to know the allocation of patients until the end of investigation and who took no part in the assessment of patients". |
| Blinding of outcome assessment (detection bias) | Low risk | "Quote: "Patients did not know that they were receiving different methods of ECT (...) Cases were alternately allocated (...) by one of us, who was the only one to know the allocation of patients until the end of investigation and who took no part in the assessment of patients". |
| Incomplete outcome data (attrition bias) | Unclear risk | No information provided. |
| Selective reporting (reporting bias) | Low risk | Data about primary outcome were reported. |
| Other bias | Unclear risk | Sponsorship bias can not ruled out. |

***Eli Lilly 2001***

| **Bias** | **Authors' judgement** | **Support for judgement** |
| --- | --- | --- |
| Random sequence generation (selection bias) | Unclear risk | Quote: "patients were allocated randomly (computer-generated system)". |
| Allocation concealment (selection bias) | Unclear risk | No information provided. |
| Blinding of participants and personnel (performance bias) | High risk | Open label study. |
| Blinding of outcome assessment (detection bias) | High risk | Open label study. |
| Incomplete outcome data (attrition bias) | Low risk | Information about data analyses was provided. |
| Selective reporting (reporting bias) | Low risk | Outcomes data were reported. |
| Other bias | High risk | Study was sponsored by Eli Lilly. (Data retrieved also from a Cochrane SR). |

***Emsley 2002***

| **Bias** | **Authors' judgement** | **Support for judgement** |
| --- | --- | --- |
| Random sequence generation (selection bias) | Unclear risk | Quote: "Subjects were randomly assigned". |
| Allocation concealment (selection bias) | Unclear risk | No information provided. |
| Blinding of participants and personnel (performance bias) | Unclear risk | Quote: "double blind". |
| Blinding of outcome assessment (detection bias) | Unclear risk | Quote: "double blind". |
| Incomplete outcome data (attrition bias) | Low risk | Information about data analyses was provided. |
| Selective reporting (reporting bias) | Low risk | Outcomes data were reported. |
| Other bias | High risk | Study was sponsored by Laxdale Ltd. |

***Emsley 2005***

| **Bias** | **Authors' judgement** | **Support for judgement** |
| --- | --- | --- |
| Random sequence generation (selection bias) | Unclear risk | Quote: "subjects were randomly assigned". |
| Allocation concealment (selection bias) | Unclear risk | No information provided. |
| Blinding of participants and personnel (performance bias) | Unclear risk | Quote: "single-blind (...) investigator-blinded". |
| Blinding of outcome assessment (detection bias) | Unclear risk | No information provided. |
| Incomplete outcome data (attrition bias) | Low risk | Information about data analysis was provided. (Quote: "we initially conducted an observed cases analysis for between-group comparisons. To assess the treatment effects over time and address the problem of missing values due to subject withdrawals, we performed 2 analyses on the intent-to-treat population. We employed a repeated-measures mixed-effects modeling approach for the primary efficacy measures and a last-observation-carried-forward approach for secondary measures"). |
| Selective reporting (reporting bias) | Unclear risk | Scores and SD on the rating scales (at follow up) were reported. |
| Other bias | High risk | Study was sponsored by Astra Zeneca. |

***Emsley 2008***

| **Bias** | **Authors' judgement** | **Support for judgement** |
| --- | --- | --- |
| Random sequence generation (selection bias) | Unclear risk | Quote: "Subjects were randomly assigned". |
| Allocation concealment (selection bias) | Unclear risk | No information provided. |
| Blinding of participants and personnel (performance bias) | Unclear risk | Quote: "12-week double-blind (...) optional open-label extension phase (...) for 40 weeks.". |
| Blinding of outcome assessment (detection bias) | Unclear risk | No information provided. |
| Incomplete outcome data (attrition bias) | Unclear risk | Information about data analysis was reported. |
| Selective reporting (reporting bias) | Low risk | Outcomes data were reported. |
| Other bias | High risk | Study was sponsored by Laxdale. |

***Erinfolami 2009***

| **Bias** | **Authors' judgement** | **Support for judgement** |
| --- | --- | --- |
| Random sequence generation (selection bias) | High risk | Authors do not use the term "randomization". Quote: "patients were included". |
| Allocation concealment (selection bias) | Unclear risk | No information provided. |
| Blinding of participants and personnel (performance bias) | Unclear risk | No information provided. |
| Blinding of outcome assessment (detection bias) | Unclear risk | No information provided. |
| Incomplete outcome data (attrition bias) | Unclear risk | No information about data analysis. |
| Selective reporting (reporting bias) | Unclear risk | No information provided. |
| Other bias | Unclear risk | No information provided. |

***Fawzi 2009***

| **Bias** | **Authors' judgement** | **Support for judgement** |
| --- | --- | --- |
| Random sequence generation (selection bias) | Unclear risk | Quote: "randomized". |
| Allocation concealment (selection bias) | Unclear risk | No information provided. |
| Blinding of participants and personnel (performance bias) | High risk | Open-label design. |
| Blinding of outcome assessment (detection bias) | High risk | Open-label design. |
| Incomplete outcome data (attrition bias) | Unclear risk | No information about data analysis (no data available). |
| Selective reporting (reporting bias) | Unclear risk | No data available. |
| Other bias | Unclear risk | Sponsorship bias can not ruled out. |

***Golden 2008***

| **Bias** | **Authors' judgement** | **Support for judgement** |
| --- | --- | --- |
| Random sequence generation (selection bias) | Unclear risk | Quote: "patients were randomized". |
| Allocation concealment (selection bias) | Unclear risk | No information provided. |
| Blinding of participants and personnel (performance bias) | High risk | Open label design. |
| Blinding of outcome assessment (detection bias) | High risk | Open label design. |
| Incomplete outcome data (attrition bias) | Low risk | Information about data analysis was reported. |
| Selective reporting (reporting bias) | Unclear risk | some outcomes data were not reported. |
| Other bias | High risk | Study was sponsored by Azur Pharma and both authors' affiliations were in Azur Pharma (US). |

***Loza 1999***

| **Bias** | **Authors' judgement** | **Support for judgement** |
| --- | --- | --- |
| Random sequence generation (selection bias) | Unclear risk | Quote: "patients were randomized". |
| Allocation concealment (selection bias) | Unclear risk | No information provided. |
| Blinding of participants and personnel (performance bias) | High risk | Open label design. |
| Blinding of outcome assessment (detection bias) | High risk | Open label design. |
| Incomplete outcome data (attrition bias) | Low risk | Information about data analysis was provided. |
| Selective reporting (reporting bias) | Unclear risk | No clear information on drop-out rates |
| Other bias | High risk | Study was performed and sponsored by Eli Lilly. |

***Makanjuola 1988***

| **Bias** | **Authors' judgement** | **Support for judgement** |
| --- | --- | --- |
| Random sequence generation (selection bias) | Unclear risk | Quote: "(Patients) were randomly allocated into 3 groups". |
| Allocation concealment (selection bias) | Unclear risk | No information provided. |
| Blinding of participants and personnel (performance bias) | Unclear risk | No information provided. |
| Blinding of outcome assessment (detection bias) | Unclear risk | No information provided. |
| Incomplete outcome data (attrition bias) | Unclear risk | No information provided. |
| Selective reporting (reporting bias) | Low risk | Primary outcome was reported. |
| Other bias | Unclear risk | Sponsorship bias can not ruled out. (Authors declared that all drugs were supplied by Dr. Manian of the National Institute of Mental Health, US). |

***Martyns-Yellowe 1993***

| **Bias** | **Authors' judgement** | **Support for judgement** |
| --- | --- | --- |
| Random sequence generation (selection bias) | Unclear risk | Quote: "The subjects were randomly assigned". |
| Allocation concealment (selection bias) | Unclear risk | No information provided. |
| Blinding of participants and personnel (performance bias) | Unclear risk | Quote: "double blind". |
| Blinding of outcome assessment (detection bias) | Unclear risk | Quote: "The assessment team itself was blind to the drug administered". |
| Incomplete outcome data (attrition bias) | Unclear risk | No information about data analysis. |
| Selective reporting (reporting bias) | High risk | SDs on BPRS scores were not reported. No clear information about "monthly clinical progress assessment". |
| Other bias | Unclear risk | Sponsorship bias can not ruled out. |

***Mechri 2006***

| **Bias** | **Authors' judgement** | **Support for judgement** |
| --- | --- | --- |
| Random sequence generation (selection bias) | High risk | Quote: "the sample was divided into two groups". |
| Allocation concealment (selection bias) | Unclear risk | No information provided. |
| Blinding of participants and personnel (performance bias) | Unclear risk | No information provided. |
| Blinding of outcome assessment (detection bias) | Unclear risk | No information provided. |
| Incomplete outcome data (attrition bias) | Unclear risk | No information provided. |
| Selective reporting (reporting bias) | Unclear risk | No information provided. |
| Other bias | Unclear risk | No information provided. |

***NCT00463372***

| **Bias** | **Authors' judgement** | **Support for judgement** |
| --- | --- | --- |
| Random sequence generation (selection bias) | Unclear risk | Quote: "Randomized". |
| Allocation concealment (selection bias) | Unclear risk | No information provided. |
| Blinding of participants and personnel (performance bias) | Unclear risk | Quote: "double-blind (investigator and subject-blind, sponsor unblinded)". |
| Blinding of outcome assessment (detection bias) | Unclear risk | No information provided. |
| Incomplete outcome data (attrition bias) | Unclear risk | No information provided. (No information about data analysis). |
| Selective reporting (reporting bias) | Unclear risk | No information provided. (No outcomes data reported). |
| Other bias | High risk | Study was carried out by Pfizer. |

***NCT00693472***

| **Bias** | **Authors' judgement** | **Support for judgement** |
| --- | --- | --- |
| Random sequence generation (selection bias) | Unclear risk | Quote: "Randomized". |
| Allocation concealment (selection bias) | Unclear risk | No information provided. |
| Blinding of participants and personnel (performance bias) | Unclear risk | Quote: "double-blind (investigator and subject-blind)". |
| Blinding of outcome assessment (detection bias) | Unclear risk | No information provided. |
| Incomplete outcome data (attrition bias) | Unclear risk | No information provided. (No information about data analysis). |
| Selective reporting (reporting bias) | Unclear risk | No information provided. (No outcomes data reported). |
| Other bias | High risk | Study was carried out by Shering-Plough. |

***NCT01172106***

| **Bias** | **Authors' judgement** | **Support for judgement** |
| --- | --- | --- |
| Random sequence generation (selection bias) | Unclear risk | Quote: "randomized". |
| Allocation concealment (selection bias) | Unclear risk | No information provided. |
| Blinding of participants and personnel (performance bias) | Unclear risk | Quote: "single blind (outcomes assessor)". |
| Blinding of outcome assessment (detection bias) | Unclear risk | Quote: "single blind (outcomes assessor)". |
| Incomplete outcome data (attrition bias) | Low risk | Quote: "ITT analysis using analysis of covariance and moderation analysis were used". |
| Selective reporting (reporting bias) | Unclear risk | Outcome data were provided. |
| Other bias | Unclear risk | No clear information provided (maybe pharmaceutical industries were not involved. This study was focused on psycho-education intervention). |

***Neppe 1983***

| **Bias** | **Authors' judgement** | **Support for judgement** |
| --- | --- | --- |
| Random sequence generation (selection bias) | High risk | No information about sequence generation. |
| Allocation concealment (selection bias) | High risk | No information provided. |
| Blinding of participants and personnel (performance bias) | Unclear risk | No information provided. |
| Blinding of outcome assessment (detection bias) | Unclear risk | No information provided. |
| Incomplete outcome data (attrition bias) | Unclear risk | No information provided. |
| Selective reporting (reporting bias) | Unclear risk | No information provided. |
| Other bias | Unclear risk | No information provided. |

***Odejide 1982***

| **Bias** | **Authors' judgement** | **Support for judgement** |
| --- | --- | --- |
| Random sequence generation (selection bias) | Unclear risk | Quote: "Assignement based on a randomized schedule". |
| Allocation concealment (selection bias) | Unclear risk | No information provided. |
| Blinding of participants and personnel (performance bias) | Unclear risk | Quote: "Patients were unaware of the contents of their injections". No other information provided. |
| Blinding of outcome assessment (detection bias) | Unclear risk | Quote: "The psychiatrist who evaluated follow-up status was blind to treatment status". No other information provided. |
| Incomplete outcome data (attrition bias) | Unclear risk | No information provided about data analysis. |
| Selective reporting (reporting bias) | High risk | Rating scales scores and SD were not reported. |
| Other bias | Unclear risk | Sponsorship bias can not ruled out. |

***Oosthuizen 2004***

| **Bias** | **Authors' judgement** | **Support for judgement** |
| --- | --- | --- |
| Random sequence generation (selection bias) | Unclear risk | Quote: "subjects were randomized 1.1 into one of the two treatment groups". |
| Allocation concealment (selection bias) | Unclear risk | No information provided. |
| Blinding of participants and personnel (performance bias) | Unclear risk | Quote: "Double-blind. (Subject) received a single, identical looking capsule per day". |
| Blinding of outcome assessment (detection bias) | Unclear risk | Quote: "Double blind". No further information. |
| Incomplete outcome data (attrition bias) | Low risk | Quote: "Analysis was conducted on the intent-to-treat population, with last observation carried forward. Outcomes data were reported". |
| Selective reporting (reporting bias) | Low risk | Outcomes measures were reported. |
| Other bias | Low risk | The study was independent from pharmaceutical industry. |

***Rompel 1978***

| **Bias** | **Authors' judgement** | **Support for judgement** |
| --- | --- | --- |
| Random sequence generation (selection bias) | Unclear risk | Quote: "The selection of cases was done on a random basis (...) allocation was done at random". |
| Allocation concealment (selection bias) | Unclear risk | No information provided. |
| Blinding of participants and personnel (performance bias) | Unclear risk | Quote: "The drugs supplied were in their ordinary commercial form and were bottled in numbered bottles with no other information (...) twenty-five cases were selected and each was given a number corresponding to that on one of the bottles (...) as the tablets were readily recognizable, they were issued by senior nursing staff in order to maintain investigator blindness; the staff were strictly forbidden to tell us, except in the case of drop-out, and in their reports they only stated what the number of tablets issued to a particular patient was and how many tablets per day were given. The numbers were in accordance with a sealed code and it did not prove necessary to break the seal during the experiment, except in the case of one of the three drop-outs, who became unmanageable". |
| Blinding of outcome assessment (detection bias) | Unclear risk | No information provided. |
| Incomplete outcome data (attrition bias) | Unclear risk | No information provided. |
| Selective reporting (reporting bias) | Low risk | Primary outcomes were reported. |
| Other bias | Unclear risk | Sponsorship bias can not ruled out. |

***Sefasi 2008***

| **Bias** | **Authors' judgement** | **Support for judgement** |
| --- | --- | --- |
| Random sequence generation (selection bias) | Unclear risk | Quote: "randomized". |
| Allocation concealment (selection bias) | Unclear risk | No information provided. |
| Blinding of participants and personnel (performance bias) | Unclear risk | No information provided. |
| Blinding of outcome assessment (detection bias) | Unclear risk | No information provided. |
| Incomplete outcome data (attrition bias) | Unclear risk | No clear information about data analysis. |
| Selective reporting (reporting bias) | High risk | Many ranting scales scores were not reported. |
| Other bias | Unclear risk | Sponsorship bias can not ruled out. |

***Shibre 2007***

| **Bias** | **Authors' judgement** | **Support for judgement** |
| --- | --- | --- |
| Random sequence generation (selection bias) | Unclear risk | Quote: "randomized". |
| Allocation concealment (selection bias) | Unclear risk | No information provided. |
| Blinding of participants and personnel (performance bias) | Unclear risk | Quote: "double blind". |
| Blinding of outcome assessment (detection bias) | Unclear risk | Quote: "double blind". |
| Incomplete outcome data (attrition bias) | Unclear risk | No information about data analysis. |
| Selective reporting (reporting bias) | Unclear risk | No information about outcomes data. |
| Other bias | Unclear risk | Sponsorship bias can not ruled out. |

***Subramaney 1998***

| **Bias** | **Authors' judgement** | **Support for judgement** |
| --- | --- | --- |
| Random sequence generation (selection bias) | Unclear risk | Quote: "Randomized". |
| Allocation concealment (selection bias) | Unclear risk | No information provided. |
| Blinding of participants and personnel (performance bias) | Unclear risk | Quote: "Double-blind". |
| Blinding of outcome assessment (detection bias) | Unclear risk | No information provided. |
| Incomplete outcome data (attrition bias) | Unclear risk | No clear information about data analysis. |
| Selective reporting (reporting bias) | Low risk | Outcomes data were reported. |
| Other bias | Unclear risk | Sponsorship bias can not ruled out. |

***Ukpong 2002***

| **Bias** | **Authors' judgement** | **Support for judgement** |
| --- | --- | --- |
| Random sequence generation (selection bias) | Unclear risk | Quote: "Consecutive individuals (...) were randomly allocated". |
| Allocation concealment (selection bias) | Unclear risk | No information provided. |
| Blinding of participants and personnel (performance bias) | Unclear risk | Quote: "double blind". |
| Blinding of outcome assessment (detection bias) | Unclear risk | Quote: "the assessor was blind to the treatment groups. the first author blindly assessed all patients before the beginning of the trial". |
| Incomplete outcome data (attrition bias) | Unclear risk | Data analysis on primary outcomes was performed only for completers. |
| Selective reporting (reporting bias) | Low risk | Primary outcomes were reported. |
| Other bias | Unclear risk | Sponsorship bias can not ruled out. |

***Uys 1996***

| **Bias** | **Authors' judgement** | **Support for judgement** |
| --- | --- | --- |
| Random sequence generation (selection bias) | Unclear risk | Quote: "patients (...) were randomized". |
| Allocation concealment (selection bias) | Unclear risk | No information provided. |
| Blinding of participants and personnel (performance bias) | Unclear risk | Quote: "double-blind". |
| Blinding of outcome assessment (detection bias) | Unclear risk | Quote: "double-blind". |
| Incomplete outcome data (attrition bias) | Unclear risk | No clear information about data analysis. |
| Selective reporting (reporting bias) | High risk | SD on rating scales scores were not reported. |
| Other bias | High risk | Study was sponsored by Lundbeck. |

***Van Wik 1971***

| **Bias** | **Authors' judgement** | **Support for judgement** |
| --- | --- | --- |
| Random sequence generation (selection bias) | Unclear risk | Quote: "Allocation to either of the three groups was at random". |
| Allocation concealment (selection bias) | Unclear risk | No information provided. |
| Blinding of participants and personnel (performance bias) | High risk | Open label study. Quote: "We could not introduce any blind procedures into the trial". |
| Blinding of outcome assessment (detection bias) | High risk | Open label study. Quote: "We could not introduce any blind procedures into the trial" |
| Incomplete outcome data (attrition bias) | Unclear risk | No information provided. |
| Selective reporting (reporting bias) | High risk | Data about rating scales scores were not reported. |
| Other bias | High risk | Study was sponsored by Wander Laboratories (Novartis). |

***Van Wik 1972***

| **Bias** | **Authors' judgement** | **Support for judgement** |
| --- | --- | --- |
| Random sequence generation (selection bias) | Unclear risk | Quote: "randomized design". |
| Allocation concealment (selection bias) | Unclear risk | No information provided. |
| Blinding of participants and personnel (performance bias) | Unclear risk | Quote: "we were aware that at least the first bottle in each box of 20 bottles contained active drug but at any point from there onwards all the succeeding bottles contained either active drug or placebo according to a randomized design, the code of which was unknown to us. Also unknown to us at the time was the fact that no bottle used after week 12 contained active drug". |
| Blinding of outcome assessment (detection bias) | Unclear risk | No information provided. |
| Incomplete outcome data (attrition bias) | Unclear risk | No clear information about data analysis. |
| Selective reporting (reporting bias) | High risk | Questionnaire scores on side effects were not reported. |
| Other bias | High risk | Study was sponsored by Ethnor Laboratories. |

***Vanelle 2004***

| **Bias** | **Authors' judgement** | **Support for judgement** |
| --- | --- | --- |
| Random sequence generation (selection bias) | Unclear risk | Quote: "patients were randomized". |
| Allocation concealment (selection bias) | Unclear risk | No information provided. |
| Blinding of participants and personnel (performance bias) | Unclear risk | Quote: "double blind". |
| Blinding of outcome assessment (detection bias) | Unclear risk | Quote: "double blind". |
| Incomplete outcome data (attrition bias) | Unclear risk | No clear information about data analysis. |
| Selective reporting (reporting bias) | Unclear risk | No available data. |
| Other bias | Unclear risk | Sponsorship bias can not ruled out. |

***Verster 1998***

| **Bias** | **Authors' judgement** | **Support for judgement** |
| --- | --- | --- |
| Random sequence generation (selection bias) | Unclear risk | Quote: "patients randomized to two parallel groups". |
| Allocation concealment (selection bias) | Unclear risk | No information provided. |
| Blinding of participants and personnel (performance bias) | Unclear risk | Quote: "double-blind study (...) the medication was supplied to the main investigator in syringes marked only by the patients' identification number and by the dosage of fluphenazine. The two products were indistinguishable in respect of colour and consistency". |
| Blinding of outcome assessment (detection bias) | Unclear risk | No information provided. |
| Incomplete outcome data (attrition bias) | Unclear risk | No information provided. |
| Selective reporting (reporting bias) | High risk | PANSS scores and CI were not reported. |
| Other bias | Unclear risk | Sponsorship bias can not ruled out. |

***Wessels 1972***

| **Bias** | **Authors' judgement** | **Support for judgement** |
| --- | --- | --- |
| Random sequence generation (selection bias) | Unclear risk | Quote: "double-blind basis". |
| Allocation concealment (selection bias) | Unclear risk | No information provided. |
| Blinding of participants and personnel (performance bias) | Unclear risk | Quote: "double blind". |
| Blinding of outcome assessment (detection bias) | Unclear risk | No information provided. |
| Incomplete outcome data (attrition bias) | Unclear risk | No information about data analysis. |
| Selective reporting (reporting bias) | Low risk | Primary outcome measures were reported. |
| Other bias | Unclear risk | Sponsorship bias can not ruled out. |
